# OncoMX: A Knowledgebase for Exploring Cancer Biomarkers in the Context of Related Cancer and Healthy Data

**DOI:** 10.1200/CCI.19.00117

**Published:** 2020-03-06

**Authors:** Hayley M. Dingerdissen, Frederic Bastian, K. Vijay-Shanker, Marc Robinson-Rechavi, Amanda Bell, Nikhita Gogate, Samir Gupta, Evan Holmes, Robel Kahsay, Jonathon Keeney, Heather Kincaid, Charles Hadley King, David Liu, Daniel J. Crichton, Raja Mazumder

**Affiliations:** ^1^ The George Washington University, Washington DC; ^2^ SIB Swiss Institute of Bioinformatics, Lausanne, Switzerland; ^3^ Department of Ecology and Evolution, University of Lausanne, Lausanne, Switzerland; ^4^ University of Delaware, Newark, DE; ^5^ NASA Jet Propulsion Laboratory, Pasadena, CA

## Abstract

**PURPOSE:**

The purpose of OncoMX^1^ knowledgebase development was to integrate cancer biomarker and relevant data types into a meta-portal, enabling the research of cancer biomarkers side by side with other pertinent multidimensional data types.

**METHODS:**

Cancer mutation, cancer differential expression, cancer expression specificity, healthy gene expression from human and mouse, literature mining for cancer mutation and cancer expression, and biomarker data were integrated, unified by relevant biomedical ontologies, and subjected to rule-based automated quality control before ingestion into the database.

**RESULTS:**

OncoMX provides integrated data encompassing more than 1,000 unique biomarker entries (939 from the Early Detection Research Network [EDRN] and 96 from the US Food and Drug Administration) mapped to 20,576 genes that have either mutation or differential expression in cancer. Sentences reporting mutation or differential expression in cancer were extracted from more than 40,000 publications, and healthy gene expression data with samples mapped to organs are available for both human genes and their mouse orthologs.

**CONCLUSION:**

OncoMX has prioritized user feedback as a means of guiding development priorities. By mapping to and integrating data from several cancer genomics resources, it is hoped that OncoMX will foster a dynamic engagement between bioinformaticians and cancer biomarker researchers. This engagement should culminate in a community resource that substantially improves the ability and efficiency of exploring cancer biomarker data and related multidimensional data.

## INTRODUCTION

Cancer biomarkers are molecules that can be assayed from bodily fluids or tissues whose presence indicates some process(es) associated with cancer.^2^ Molecular characterization of cancers can lead to pan-cancer or cancer type–specific biomarkers, which are becoming integral components of risk assessment, pathologic diagnosis, monitoring of disease progression, and therapeutic decisions.^3,4^ This is especially true when biomarker assays enable the identification of subpopulations amenable to treatment by targeted molecular therapies. Cancer biomarkers are commonly individual genes or proteins, multigene/protein panels, or biomolecules, like glycans, analyzed from urine, blood, stool, or other biologic sample. Biomarker diversity is ever growing and may also include findings from image analyses,^5^ gut microbiome abundance,^6^ and others.

Attempts at molecular characterization of cancer samples have increased as a result of advances in high-throughput sequencing and other technologic progress.^7,8^ Paired with increased funding opportunities, such as those enabled through the Precision Medicine Initiative of 2015, it is not surprising to find increased reports of potentially novel cancer biomarkers.^9^ However, reproducibility of initial findings, clinical validation, and access to harmonized biomarker and associated data remain formidable challenges.^7^

While issues of reproducibility and clinical validation must be addressed by biomarker research and regulatory communities, usability and accessibility of findings can be addressed in the public domain. In fact, a number of research groups already make cancer biomarker data available, including EDRN—the Early Detection Research Network focusing on the research and development of biomarkers and technologies for the clinical application of early cancer detection strategies^10^; CBD—the Colorectal Cancer Biomarker Database containing colorectal cancer biomarkers reported from articles in PubMed;^11^ ResMarkerDB—the database of biomarkers of drug response to monoclonal antibody therapy in breast and colorectal cancer,^12^ and more.^13^ Additional current and past efforts exist, such as those belonging to the Cancer Biomarkers Research Group of the National Cancer Institute Division of Cancer Prevention, including the Alliance of Glycobiologists for Cancer Research,^14^ the Consortium for Imaging and Biomarkers (CIB),^15^ and others,^16^ and more biomarker information can be obtained directly from the literature or other published sources. Although not always biomarker centric, the National Cancer Institute Informatics Technology for Cancer Research (ITCR) program funds a number of resources, including HemOnc,^17^ the Cancer Proteome Atlas (TCPA),^18^ the Patient-Specific Drug-Gene Networks for Recommending Targeted Therapies (CDGNet),^19^ and others, that describe, generate, analyze, or link cancer data that could provide additional evidence for biomarker relevance. It seems logical, then, that these various data could be combined into a meta-resource for the search and exploration of cancer biomarker–related information.

**CONTEXT**

**Key objective:**
To develop a data model and central, unified, integrated Web resource to enable improved exploration of cancer biomarkers in the context of related evidence.
**Knowledge generated:**
A data model was developed to describe cancer biomarker evidence and that was capable of accommodating heterogeneously structured extant data and extensible to diverse new data types. Data integrated and unified through this model were made available through OncoMX, a knowledgebase and Web portal for exploring cancer biomarker data and related evidence.
**Relevance:**
Cancer biomarker evidence can be accumulated across various resources from a single access point through OncoMX to generate summary reports for a given gene, including available information on clinical status and relevance. Furthermore, OncoMX is linking to several other resources, improving the reach of each resource reciprocally and increasing the utility of any given data set.

However, the above-mentioned data sets are highly variable in scope, developed for specific biological applications, specific to certain cancer types, limited by regulatory status, or pertinent to specific clinical populations. Furthermore, data formats are frequently heterogeneous, lacking common attributes to enable integration. Improved modeling of cancer biomarker data would facilitate the integration of interscope biomarker data. Ontology unification and mapping through common resource accessions would improve the quality of integration, downstream assertions, and reasoning. Improved data provenance tracking would transparently communicate parametric configurations and assumptions made during processing and retrieval, and the availability of integrated data (searchable via Web portal designed with key user input) would enhance the usability of the underlying biomarker data.

To address these issues, the OncoMX group developed a data model to integrate public biomarker data from EDRN and the US Food and Drug Administration (FDA) and additional related data around persistent accessions and identifiers. The resulting model provides the foundation for integration of heterogeneous biomarker evidence (using the BioCompute Object [BCO] framework^20,21)^) into the OncoMX knowledgebase and Web portal for cancer biomarker exploration. Integrated data types include cancer mutation, cancer differential expression (mRNA and miRNA), cancer expression specificity (single cell RNA [scRNA] sequencing), healthy expression (mRNA from human and mouse), literature mining for cancer mutation and expression, EDRN biomarkers, and FDA-approved breast cancer biomarkers^2^.

## METHODS

**FIG 1.**
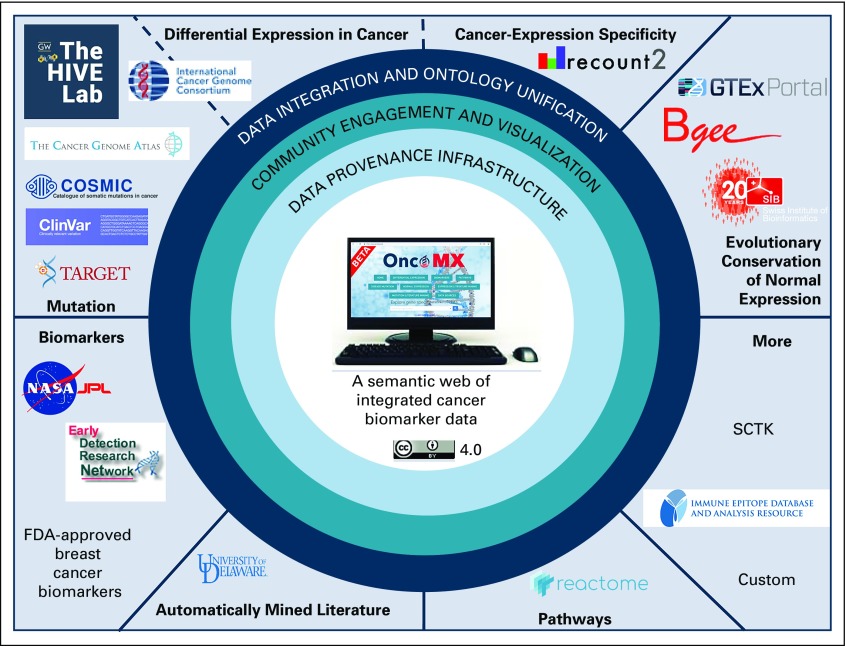
OncoMX overview. External segments: Data for cancer mutation, cancer expression, healthy expression, literature mining for both cancer mutation and expression, cancer biomarkers, relevant ontologies, functional annotations, and pathways were initially harvested from a number of publicly available resources, such as The Cancer Genome Atlas, International Cancer Genome Consortium, ClinVar, COSMIC, UniProt, and others, and further processed or analyzed by The George Washington University for BioMuta and BioXpress; SIB (Swiss Institute of Bioinformatics) for Bgee (RNA sequencing–derived healthy expression calls and ranks for human and mouse); the University of Delaware for literature mining through DiMeX and DEXTER; and the Early Detection Research Network for biomarkers. Outer ring: Data sets were integrated and unified through Cancer Disease Ontology slim terms for disease names and Uberon Anatomical Entity terms for tissue and physiologic location. Middle ring: Feedback was solicited through a multipronged approach involving use case collection at poster sessions and two formal workshops. With user community guidance, a series of data views and graphic visualizations were devised to aid end users in the exploration and interpretation of biomarker evidence. Inner ring: Data sets were refined and documented with provenance details following the BioCompute Object model for data provenance capture and hosted through the data.oncomx.org data site. Inner circle: The product of these efforts is the current OncoMX Web portal, a semantic web of integrated cancer biomarker data that is readily accessible and licensed under a Creative Commons Attribution 4.0 International License. FDA, US Food and Drug Administration; SCTK, Single-Cell Toolkit.^40^

Figure 1 shows an overview of OncoMX integration.

### Data Retrieval

Data were provided as .txt, .tsv, or .csv files for the following: literature mining for differential expression in cancer from DEXTER—Disease-Expression Relation Extraction from Text^22^ and mutation in cancer from Extraction of Mutation Association to Diseases (DiMeX) (University of Delaware)^23^; RNA sequencing–derived healthy expression calls and ranks for human and mouse from Bgee, a Database for Gene Expression Evolution (SIB Swiss Institute of Bioinformatics)^24^; and the FDA-approved breast cancer biomarkers and cancer cell–type expression specificity from scRNA sequencing (George Washington University). Data were pulled from external consortium databases for public biomarker data from EDRN,^10^ cancer mutation data from BioMuta,^25,26^ cancer differential expression of mRNA and miRNA from BioXpress,^26,27^ and Reactome.^28^ The Data Supplement provides a summary of data access and pertinent details for OncoMX and contributing resources.

### Processing and Unification

Many data sets used by OncoMX were initially processed by external pipelines documented in source resources. A summary of relevant preprocessing details for cancer mutation, cancer differential expression, healthy expression, and biomarkers is included in the Data Supplement, along with links to documentation. Data sets were unified through Cancer Disease Ontology (CDO) slim^29^ terms for disease names and through Uberon Anatomical Entity Terms^30^ for anatomic structure names. Data not already annotated with the UniProt Knowledgebase (UniProtKB)^31^ accessions (AC) were mapped to the canonical set of UniProtKB AC through gene symbol, transcript, biomarker name, or genes included in a biomarker panel, and AC were filtered if existing outside the canonical set. An OncoMX field dictionary was created that leveraged field names and ontology terms of existing resources (UniProtKB, GlyGen,^32^ HUGO Gene Nomenclature Committee (HGNC),^33^ and so on), and headers were uniformly formatted and mapped to existing OncoMX field names. Resulting files were checked for completeness and adherence to expected content and subjected to automated quality check to ensure integrity and format sanity. Pipeline steps, provenance details, and other metadata were captured using the BCO^20^ specification and output in JavaScript Object Notation (JSON) and .txt formats. All processed data and corresponding metadata objects were entered into a database and subjected to version control. Additional processing considerations for specific data sets are described below.

#### Mutation and differential expression in cancer.

Earlier versions of the cancer mutation and differential expression data sets were updated to include additional data (from The Cancer Genome Atlas, International Cancer Genome Consortium, and Clinical Interpretations of Variants in Cancer [CIViC]), and the expression data set was updated to include miRNA analysis.^26^ A new pipeline was developed for cancer mutation to improve the tracking of downloaded content, unify data in .vcf format, subject resulting information to automated quality check, automate mapping of disease terms to CDO slim terms and genes/transcripts to UniProtKB AC using gene symbols or alternate accessions, generate frequency across sources, and automate site annotation and functional impact prediction. Minor updates were made to the differential expression analysis pipeline, including updating the analysis package to DESeq2,^34^ mapping cancer subtypes to parent CDO slim terms, and quantifying patient ratios for each direction of expression change (the Data Supplement provides additional details). Resulting data were subjected to the general OncoMX processing steps outlined above before integration.

#### Healthy expression.

A custom format of healthy (wild type) expression data was devised for RNA sequencing–derived expression calls for human and mouse. Data were limited to tissue types that had at least one reported association in the cancer mutation or differential expression data sets, as determined through mapping between CDO slim and Uberon Anatomical Entity. Data were further limited to adult developmental life stages. Analyzed expression values were ranked in two series for each species: one compared the expression of a given gene with that of all genes in a given tissue and the other compared the expression of a given gene with that of the same gene across all tissues. To enable cross-species exploration of expression profiles, the set of 1:1 orthologs between human and mouse were retrieved from the Orthologous MAtrix (OMA) database^35^ and used to map human genes to orthologous mouse genes (the Data Supplement provides additional details).

#### Literature mining.

DEXTER,^22^ an automated text-mining tool, was applied on Medline abstracts to extract gene, disease, expression level, experimental context, and conditions being compared. Customized for OncoMX, DEXTER extracted expression differences in cancer compared with normal/control tissues and verified whether the normal tissue came from the same patient or not. DEXTER was applied on a comprehensive set of cancer-related abstracts identified using the PubMed query “cancer OR cancers OR carcinoma OR carcinomas OR neoplasm OR neoplasms,” which returned 3,717,745 abstracts (as of March 2018). Abstracts were filtered for those that contained words or phrases pertinent to expression, which reduced the number of abstracts to 1,750,928.

DiMeX^23^ is a tool that detects different types of connections between mutations and disease in the literature. For OncoMX, less reliable connections, such as those extracted on the basis of comentions, were dropped while maintaining association relations between mutation and a disease aspect. The mutation detection module of DiMeX was improved by integration with tmVar^36^ after performance evaluation of several mutation detection tools on a range of well-known corpora. The resulting integration enabled the reduction of false negatives without increasing false positives. DiMeX’s relation extraction module was also refined and improved by integrating more recent parsing technology.

#### Cancer biomarkers.

Public biomarker records were retrieved from EDRN, and a new data set of FDA-approved biomarkers in breast cancer was generated by manual search across Web resources (details are provided in the Data Supplement). The generation and integration of these data sets enabled the OncoMX team to troubleshoot issues of extension, facilitated augmentation of infrastructure to ensure interoperability between data of differing types, and demonstrated the immediate utility of newly added data and views available to the end user. A new, easily extensible, biomarker-centered data model was developed to describe a biomarker evidence data object, accounting for the relationships between data sets reporting clinical status of known and/or actively studied biomarkers (EDRN and/or FDA) and other biomarker evidence data types.

#### Cancer expression specificity.

Expression of 10 cancer cell types across the brain, lung, and colon were analyzed from scRNA sequencing data from three studies retrieved from recount2^37^ and integrated into OncoMX (details are provided in the Data Supplement). Count tables were filtered for low-quality cells and low-abundance genes by filtering out samples with library sizes and features that expressed fewer than three median absolute deviations from the median value of each quality control metric computed from mitochondrial gene expression across all samples. Cell-specific biases between samples were normalized with a deconvolution approach^38^ in which pooled counts from multiple samples are used to compute size factors that are deconvolved to infer size factors for each sample. Data sets were filtered for target cell sequencing runs, and dimensionality reduction was performed using principal component analysis (PCA), t-distributed stochastic neighbor embedding, and uniform manifold approximation and projection (UMAP). scRNA sequencing gene-level count matrices were analyzed for cell type–specific expression using Preferential Expression Measure (PEM), a metric designed for tissue-level expression specificity analysis,^39^ and qualitative annotations of specificity were computationally determined for each PEM score. Resulting data were mapped to UniProtKB AC, HGNC gene IDs, Ensembl gene IDs,^41^ DOIDs, and DO names, and were subjected to OncoMX processing steps described above.

### Integration and Data Modeling

All data were mapped to UniProtKB AC directly or through HGNC gene ID and/or Ensembl gene ID to UniProtKB AC. A data model describing a biomarker evidence object was devised such that an object of type biomarker evidence can be diverse (molecular characteristic, clinical relevance, descriptive literature, and so on) and is currently expected to map to information about one or many genes.

### Database Architecture, Back-End Infrastructure, and Front-End Implementation

OncoMX was developed within the virtual machine environment, configured using a 32-GB ram and 12 CPU virtual machine running under the CentOS Linux 7 operating system. The database was built under the MariaDB version 5.5.60 engine, running under the same server as and connected directly to the application, developed using Python Django framework integrating bootstrap 3 for user interface design and jQuery for interactive capabilities. The application was designed with user login security based on the Django authentication library and built to use RESTful Web services to expose the underlying database for external access. The application environment, including the Django framework,^42^ was dockerized into a container, which allowed easy transfer and deployment across servers while maintaining a consistent architectural environment.

## RESULTS

### Integrated Data

Resulting data sets encompassed 939 and 96 unique biomarkers from EDRN and FDA, respectively, mapped to 20,576 genes with available mutation and/or differential expression in cancer. Sentences reporting mutation in cancer were extracted from 14,360 PubMed publications, and those reporting differential expression in cancer were extracted from 25,865 publications. Healthy expression data for 33,753 human and 45,879 mouse Ensembl gene IDs were retrieved, mapping to 19,555 canonical human genes, 15,349 with mouse orthologs. Table 1 lists data sets currently available at https://data.oncomx.org.

**TABLE 1.**
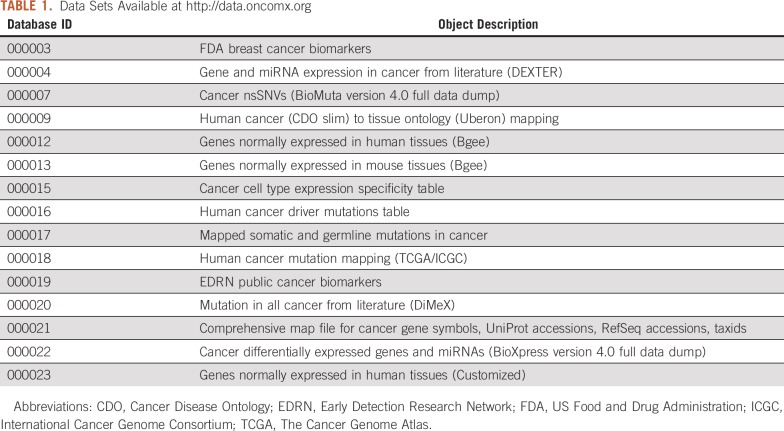
Data Sets Available at http://data.oncomx.org

### Biomarker Evidence Data Modeling

**FIG 2.**
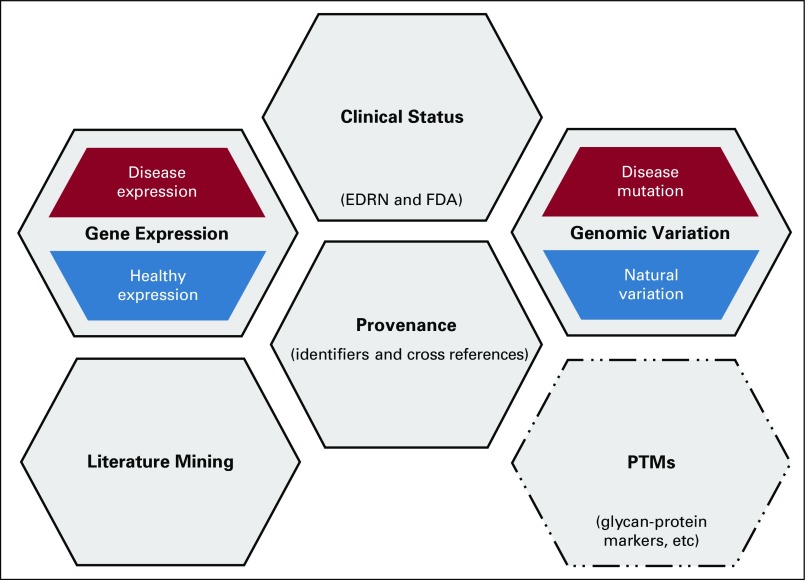
Biomarker evidence data model. This diagram shows the modular extensibility and current configuration of the biomarker evidence data model developed for OncoMX. The central provenance domain component captures biomarker and related identifiers for keys and core attributes, as well as cross references between various dictionaries. The genomic variation component contains both mutations in cancer and natural polymorphism data. The expression component also has two subcomponents to describe data coming from diseased and healthy samples. The healthy subcomponent has an added layer of organism, and both expression subcomponents can be further broken down on the basis of the experimental strategy from which data were generated (not shown). The literature mining component contains data extracted from abstracts and full articles reporting biomarker activity, and the clinical status component contains data from Early Detection Research Network (EDRN) and US Food and Drug Administration (FDA) describing clinical attributes, including approved and actual indications, status of clinical trials, related publications, and more. Of note, extension to a new evidence type, glycan-protein type biomarkers, is actively underway, which should allow for the future extension to other subtypes of the anticipated post-translational modification domain.

Integration of EDRN and FDA data sets solidified the need to describe both clinical assertions related to actively characterized cancer biomarkers along with other information that could be leveraged as biomarker evidence. A data model was developed to describe a biomarker evidence object that is extensible to any number of components but currently has components for provenance, genomic variation, gene expression, clinical status, and literature mining. Although not required, the current core of the provenance domain is the gene symbol as available biomarker related data contain or link to one or many genes or miRNAs. Granularity of biomarker-level details was retained without sacrificing gene-level detail for panels that contained multiple genes by mapping to each unique gene. The resulting model (Fig 2) accommodates all OncoMX data types and is readily extensible to new data types containing some combination of keys and additional core attributes of disease and tissue; data types not mapped to genes can still be connected through other core attributes.

### Usage and Utility

#### OncoMX Web site features.

Early Web development focused on basic gene search functionality such that a string search query results in the display of all visual and tabular information for a specified gene. Combinatorial filtering and analysis on the integrated data layer allow filtering by *P* value thresholds or specific disease labels. Other features include a landing page with search bar and quick links for data set exploration; a dashboard for visualizing statistics and summaries; a table viewer rendered dynamically on the basis of user interaction; various types of user documentation; and a contact form to solicit user feedback.

#### Use case 1: Search for an individual gene biomarker.

**FIG 3.**
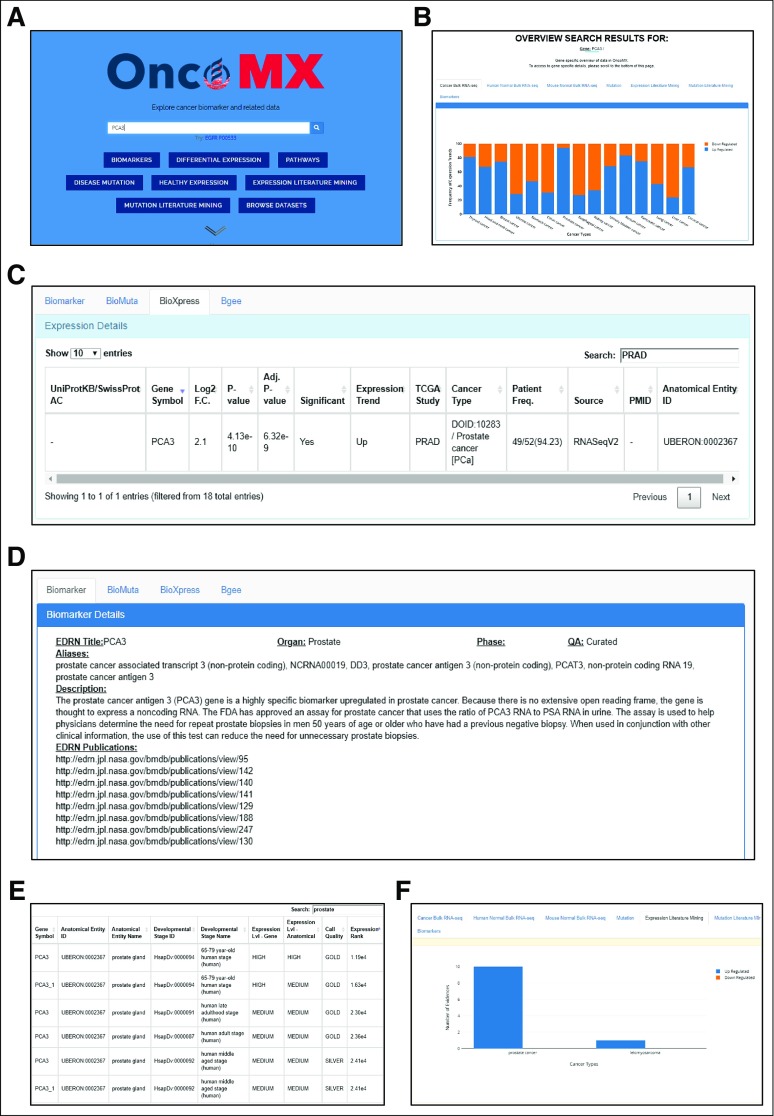
Exploring OncoMX for an individual gene biomarker: Findings for *PCA3* in prostate cancer. (A) The OncoMX landing page provides quick access to the search bar. Entering “PCA3” will redirect the user to the search results page. (B) The default open tab of the top viewer in the search results page shows mRNA differential expression results for the queried gene across all cancers. *PCA3* is shown to be upregulated in 94% of prostate cancer tumor samples compared with the corresponding adjacent normal samples. (C) Tabular and text details are also available in the lower viewer on the search result pages. Exploring the quantitative information shows that 49 of 52 samples have a logtwo-fold change increase of expression in tumor samples compared with the adjacent normal, and that the overexpression reported for prostate cancer is statistically significant. (D) Text details, including a list of biomarker aliases, a brief description, and a list of links to publications reported by Early Detection Research Network (EDRN), are available from the Biomarkers tab in the lower viewer. (E) Healthy expression of *PCA3* is found to be “MEDIUM” in earlier human adult stages but “HIGH” in the 65- to 79-year-old human stage. (F) Automatic literature mining finds 16 unique sentences from 15 unique publications reporting overexpression of *PCA3* in prostate cancer and another sentence reporting overexpression of *PCA3* in leiomyosarcoma. FDA, US Food and Drug Administration; PSA, prostate-specific antigen; RNA-seq, RNA sequencing; TCGA, The Cancer Genome Atlas.

*PCA3* is a long, noncoding RNA that is overexpressed in most prostate cancer PCa cells and is involved in regulating the expression of epithelial–mesenchymal transition markers, androgen receptor cofactors, and PCa suppressor *PRUNE2*.^43-45^ As depicted in Figure 3, search for “PCA3” shows an overexpression of *PCA3* in prostate cancer. In fact, 49 of 52 samples have a logtwo-fold change increase in *PCA3* expression in the tumor sample compared with its adjacent normal. Biomarker description, aliases, and publications reported by EDRN can be viewed in the biomarker details tab. Moreover, there are 14 literature evidences reporting upregulated *PCA3* in prostate cancer. Of note, *PCA3* expression in healthy samples is qualified as “MEDIUM” for earlier adult stages, but “HIGH” for later stages, which implies an association between *PCA3* expression and age.

#### Use case 2: Search for a biomarker panel.

**FIG 4.**
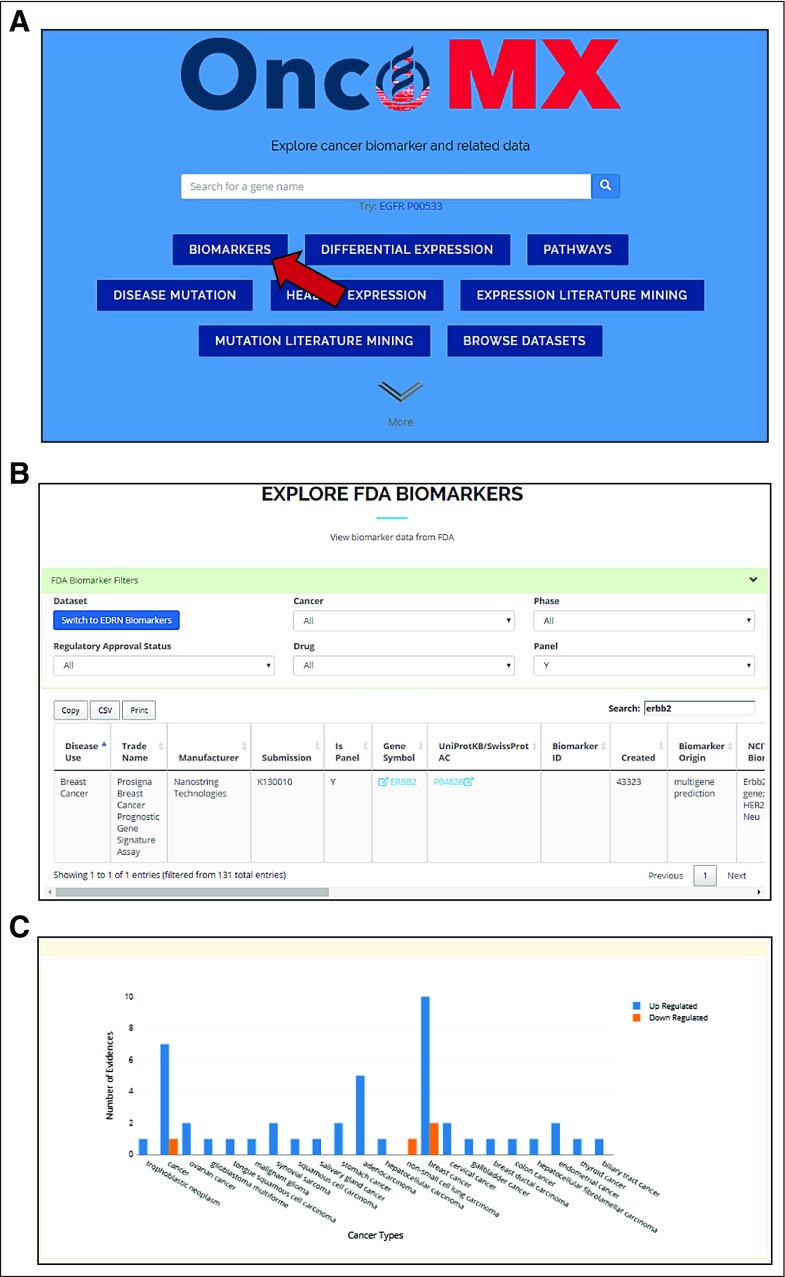
Exploring OncoMX for a gene panel biomarker: Findings for *ERBB2* as part of the Prosigna multigene prediction panel for breast cancer. (A) In addition to the search bar, the OncoMX landing page contains buttons that are quick links to other tables and views. Clicking Biomarkers will redirect the user to the biomarker exploration table viewer. (B) From the data exploration table, the user can click the arrow in the top right of the Biomarker Filters box to access the dropdown menu for filters. Clicking Switch to FDA Biomarkers will reload the table with the US Food and Drug Administration (FDA)–approved biomarker data set. Searching for “ERBB2” in the search bar immediately above the table will reload the table for hits to *ERBB2* (10 hits). Accessing the filters again, the user can search for hits belonging only to panels by selecting Y from the Panel filter dropdown. The table will once again reload to display the single hit for *ERBB2* in a panel, identifying it as part of the Prosigna multigene prediction panel for breast cancer. (C) Going back to the landing page and performing a gene search for “ERBB2” will redirect the user to the detailed search results page. Navigating to the Expression Literature Mining tab, one can readily see the standout peak indicating multiple literature evidences (n = 262) for upregulation of *ERBB2* in breast cancer, including 253 unique sentences from 248 publications. EDRN, Early Detection Research Network.

The Prosigna Breast Cancer Prognostic Gene Signature Assay panel includes 58 genes that can be used to assess a patient’s risk of disease recurrence (https://www.nanostring.com/diagnostics/prosigna/technology/prosigna-algorithm). Clicking on the Biomarkers button from the landing page will redirect the user to the data exploration table that can be toggled between biomarker data sets and filtered for various attributes. Search for “ERBB2” in this table shows that this gene, well known for its indications for treatment and prognosis in breast cancer,^46-48^ is included in 10 FDA-approved biomarker products—nine individual amplification markers and one panel, the Prosigna multigene prediction panel. In the detailed search view, there are multiple evidences for *ERBB2* in the literature, both for differential expression and mutation. Of interest, whereas the majority of differential expression evidences (n = 48) indicate upregulation, there are two evidences for downregulation. Exploration of the underlying data shows that downregulation has been observed in response to pertuzumab treatment in mouse xenograft studies^49^ and has been observed to be lower in obese patients with early-stage breast cancer.^50^ Visualization of available information shows that this gene is approved for various indications, including prognosis, prediction, and companion diagnosis (Fig 4). The Data Supplement describes additional use cases.

## DISCUSSION

### Leveraging Existing Resources to Promote Sustainability

OncoMX was designed to combine existing and newly generated data, establishing a strong application customized for cancer biomarker research. Although other multidimensional, integrated cancer resources exist, such as the (cBioPortal)^51,52^ and CIViC,^53^ the focus on biomarkers, foundational inclusion of large-scale literature mining, ontology-driven unification, and cross comparison of analysis of healthy samples across species are aspects that are unique to OncoMX. However, OncoMX leverages the utility of such extant related resources, including cBioPortal for Cancer Genomics (cBioPortal),^51,52^ CDGnet (CANCER Drug Gene Network),^19^ CDSA (Cancer Digital Slide Archive),^54^ CIViC,^53^ HemOnc,^17^ iPTMnet,^55^ PDX (patient-derived tumor xenograft) finder,^56^ and TCPA (The Cancer Proteome Atlas). This model promotes the extensibility and sustainability of OncoMX and referenced resources by allowing OncoMX to focus on harmonization, integration, annotation mapping, evidence tagging, and database maintenance while outsourcing data set creation and curation to collaborators and other experts.

### Future Directions

OncoMX is actively seeking new data and types, such as imaging, glycan biomarkers, drug targets, methylation, alternative splicing, and more. Work in progress includes extending the data model to new types, integrating FDA data sets for additional cancers upon user request, improving healthy expression and cross-species visualization, and expanding cross references to key cancer resources. The OncoMX team will continue hosting workshops to ensure that development is shaped by users, facilitating engagement between stakeholders and ultimately resulting in an adaptable resource for the improved exploration and potential discovery of cancer biomarkers.
